# Staphylococcus pseudintermedius: an undocumented, emerging pathogen in humans

**DOI:** 10.3205/dgkh000367

**Published:** 2020-12-02

**Authors:** Suneel Bhooshan, Vikrant Negi, Prabhat K. Khatri

**Affiliations:** 1 Department of Microbiology Dr. S. N. Medical College, Jodhpur, Rajasthan, India; 2 Department of Microbiology, Government Medical College, Haldwani Nainital, Uttarakhand, India

**Keywords:** Staphylococcus aureus, Staphylococcus pseudintermedius, zoonosis

## Abstract

The first infections of methicillin-resistant *Staphylococcus pseudintermedius* in humans were recorded in 2006, and is now becoming a concern because of its close similarities to human pathogens in the *Staphylococcus* *intermedius* group (SIG). These bacteria have all the properties which a multidrug-resistant *Staphylococcus aureus* possesses.

The literature was searched using the term “*Staphylococcus pseudintermedius*” in PubMed and other reference databases. The virulence factor and the pathogenicity are under investigation, but reports have suggested that this commensal of animals is transmitted easily via close contact to animals by owners, veterinarians and staff.

Resistance to beta-lactams (including methicillin) is a primary concern. Drug resistance to methicillin is a considerable problem in developing countries, as antibiotic use is not regulated. Studies from Europe have reported multidrug resistant isolates from clinical specimens. Although data on drug resistance and pathogenesis of *S. pseudintermedius* are not sufficient, it is extremely important to identify the pathogen correctly. Only then can its pathogenesis be studied during the course of disease and appropriate measures developed to prevent it becoming a global problem.

## Zusammenfassung

Der Methicillin resistente *Staphylococcus pseudintermedius*, über den Infektionen beim Menschen erstmalig 2006 berichtet wurden, gibt wegen der großen Ähnlichkeit zu humanpathogenen Krankheitserregern aus der *Staphylococcus* intermedius Gruppe (SIG) Anlass zur Besorgnis. Das Bakterium hat alle Eigenschaften eines multiresistenten *Staphylococcus aureus*.

Die Literatur wurde mit dem Begriff *„Staphylococcus pseudintermedius*“ mit der PubMed-Literaturrecherche und anderen Datenbanken durchgeführt. Derzeit werden der Virulenzfaktor und die Pathogenität untersucht. Aus Berichten geht hervor, dass der Kommensale von Tieren durch engen Kontakt auf Halter, Tierärzte und deren Personal leicht übertragen wird. Die Resistenz gegenüber Beta-Laktamen, zu denen auch Methicillin gehört, ist ein Problem.

Die Resistenz gegen Methicillin ist vor allem in den Entwicklungsländern ein Problem, in denen der Einsatz von Antibiotika nicht reguliert ist. Studien aus Europa haben über Isolate mit Mehrfachresistenz aus klinischen Proben berichtet. Obwohl es noch keine ausreichenden Daten über Antibiotikaresistenz und Pathogenese gibt, ist es äußerst wichtig, den Erreger korrekt zu identifizieren, damit die Pathogenese im Krankheitsverlauf untersucht werden kann, bevor sich ein weltweites Problem entwickelt.

## Introduction

The genus *Staphylococcus* is currently divided into 38 species and 17 subspecies. It is infamous for its drug resistance and multiple pathogenic factors [1]. Based on the presence of coagulase enzyme, genera were broadly divided in two categories: coagulase-positive and coagulase-negative species. Initially, only *Staphylococcus aureus* were thought to be a human pathogen, but in 1976. *Staphylococcus* *intermedius*, a new coagulase-positive species, was identified and reported to be associated with animal and human infections [2]. *Staphylococcus intermedius* was first considered to be a single species. Later, based on 16S rRNA typing, it was reclassified as *Staphylococcus intermedius* group (SIG), including three species: *S. intermedius*, *Staphylococcus pseudintermedius* and *Staphylococcus delphini*, which were closely related in terms of biochemical reactions. In this group, only *S. intermedius* was considered to be pathogenic in humans. *S. pseudintermedius* and *S. delphini* are canine commensals or opportunistic pathogens associated with skin and wound infections, predominately in animals. In recent veterinary literature, *S. pseudintermedius* is one of the important pathogens of zoonotic origin that causes wound and skin infections. According to the literature, up to 90% of healthy dogs may be colonized with *S. pseudintermedius* [3], [4]. *S. pseudintermedius* mimics *S. intermedius* phenotypically, which makes its identification difficult using automated identification systems. Until the last decade, it was falsely reported to be *S. intermedius* by phenotypic and automated systems, owing to a great paucity of data available for identification. Not all commercially available identification systems are able to correctly identify *S. pseudintermedius*. 

The unjustified use of antimicrobials in companion animals is responsible for emerging antimicrobial resistance. *S. pseudintermedius* is another link in the same chain in emerging drug resistance, as it is reported to be multidrug resistant, able to transmit from animals to humans, and possesses all the virulence factors of *S. aureus*. 

In 2006, the first cases of *S. pseudintermedius* infection in humans were reported by Van Hoovels [5] from 60-year-old patients with clinical presentation of ischemic cardiomyopathy and ventricle tachycardia, but it has likely been present in the community for far longer. Since then, there have been attempts to isolate and categorize this pathogen to study its virulence factors and pathogenesis in humans [5], [6]. The spectrum of infections caused by *S. pseudintermedius* is very close to *S. aureus* infections. A case series of 24 isolates by Somayaji in 2016 shows comorbidity factors, with the elderly being more prone to infection [7]. Only 2 patients (8%) out of 24 were below age 40, out of which one had a wound infection related to a dog bite.

## Methods

Using the keyword “*Staphylococcus pseudintermedius*”, we searched PubMed, finding total of 339 publications including both veterinary and human medicine, out of which 72 were reported from humans. The search also included google and public health agency information (National institutes of health [NIH)], Centers for Disease Control and Prevention [CDC], the European Centre for Disease Prevention and Control [ECDC], the US Food and Drug Administration (FDA), Agency for Healthcare Research and Quality [AHRQ], etc). We reviewed all literature published including research articles, original articles, review articles and case reports from human and veterinary medicine through July 21, 2018. The search strategy included only English-language publications.

## Results

### Genetic characterization

Several molecular methods are used for differentiating *S. pseudintermedius* from the *Staphylococcus intermedius* group (SIG, *S. intermedius, S. pseudintermedius*, and *S. delphini*), but these are limited to research purposes owing to its cost and lack of clinical association with disease. Ribotyping and PFGE are some of the various DNA-based techniques are used for *S. pseudintermedius* typing and epidemiological surveillance [8], [9], [10], [11], [12], [13]. In recent research-based studies, PCR-RFLP, spa typing and MLST are also used for typing [14], [15], [16], [17]. MALDI-TOF MS has shown promising results in identification and differentiation of SIG, although the sensitivity and specificity are not better for *S. intermedius* than for *S. pseudintermedius* [18]. Focusing on antibiotic resistance, multiplex PCR and SCC mec gene typing have been studied for macA gene detection, which is responsible for methicillin resistance. 

### Biochemical identification

*S. pseudintermedius* must be differentiated from other coagulase-positive *Staphylococcus* species by using a combination of biochemical tests (Table 1 (Tab. 1)). On blood agar plate, it shows creamy white colonies with beta-hemolysis. The lack of biochemical and automation resources to differentiate between coagulase producing species of *Staphylococcus* group usually leads to erroneous reporting of all coagulase producing species as *Staphylococcus aureus*.

*Staphylococci* are grouped together as *S. aureus*. The arginine dihydrolase test, β-gentibiose test, D-mannitol and polymyxin B disk differentiation tests are important biochemical assays which can differentiate *S. pseudintermedius* from other closely related *Staphylococci* [5], [18], [19], [20].

### Pathogenic factor and pathogenesis

Pathogenic factors are very similar to *S. aureus*. Knowledge about pathogenesis of *S. pseudintermedius* is very limited in the case of strains originating from humans. Enzymes and toxins produced by *S. pseudintermedius* have shown same activity in *in vitro* tests (Table 2 (Tab. 2)).

Panton-Valentine leukocidin of *S. aureus* is a cytotoxin that destroys leukocytes and causes tissue necrosis. A similar toxin, bio-component leukotoxin Luk-I, encoded by two genes, *lukS/F*, is also produced by *S. pseudintermedius*. Pathogenesis in humans has not been thoroughly studied and requires more detailed investigation. 

*S. pseudintermedius* is an opportunistic pathogen. It is part of the normal flora of most dogs and does not cause any disease, unless the resistance of the host is lowered and the skin barrier altered by predisposing factors, such as atopic dermatitis, medical and surgical procedures, and or immunosuppressive disorders. Similar to *S. aureus* infection in humans, colonization is likely to be a risk factor for infection and, in most circumstances, dogs are likely to become infected with a strain that they carry on their body [21], [22], [23], [24], [25], [26], [27], [28], [29], [30], [31], [32], [33], [34], [35], [36], [37], [38], [39], [40], [41], [42], [43], [44], [45], [46], [47], [48], [49], [50], [51], [52], [53], [54], [55], [56], [57], [58]. Case reports from implant devices have yielded alarming results about its pathogenesis, which suggests biofilm formation. *Staphylococcus* genera are well-known for their biofilm-forming properties. Pomilio 2015 [59] conducted an interesting and novel *in vitro* study to demonstrate biofilm formation properties by providing a simulated environment similar to wound infection by adding serum, adjusting pH and antibiotic concentrations for 48 to 72 hours of exposure. The results of that study demonstrated the ability to form biofilm *in vitro* for the first time. Along with these findings other properties were also noted, such as the effect of serum and production of abundant amounts of extracellular polymeric substance (EPS) matrix, as observed by scanning electron microscope. This simulation suggested that this bacterium can produce biofilm on implant devices such as catheters, and is able to survive in wound environments by producing excessive amount of EPS, which stops antibiotic penetration in to biofilm.

### Epidemiology

**Figure 1:**
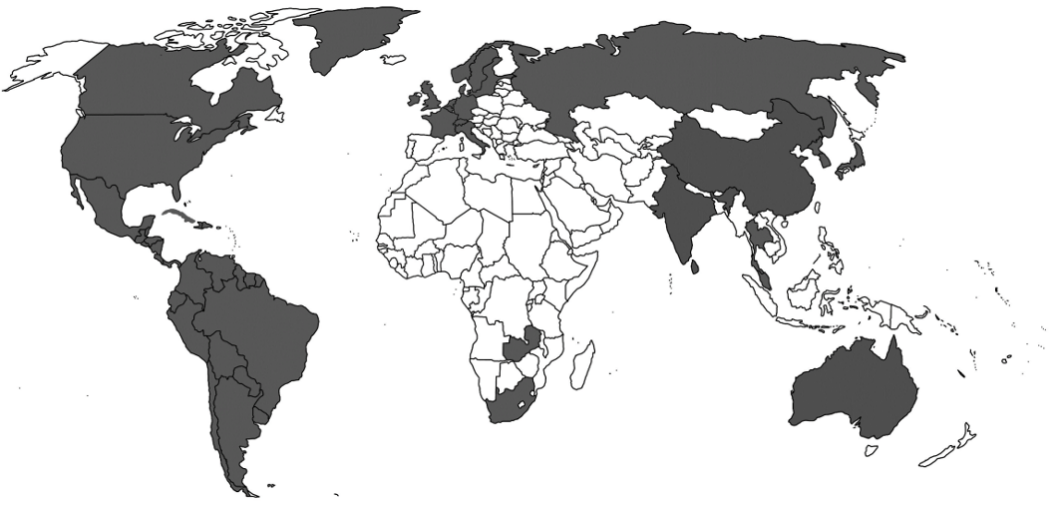
Global demographic data (reported cases in grey)

*S. pseudintermedius* was initially misdiagnosed as *S. intermedius* due to lack of data. Many details are still not available about epidemiology, transmission and risk factors, although on the basis of genetic linage, it has now been confirmed world-wide. It was first reported from Belgium, and later in other countries with different signatures in their genetic makeup when categorized by multilocus sequence types (MLST) and spa types (Figure 1 (Fig. 1), Table 3 (Tab. 3)). It is a part of normal flora in canines, colonizing the mouth, nose, perineum and groin. The transmission route is vertical in animals and horizontal or interspecies in the case of veterinary staff and dog owners via close contact with colonized pets. Risk factors in humans are immunosuppressed status, postsurgical infections, and old age. So far, there is no evidence of transmission of this pathogen between humans to human [20], [25], [58], [60], [61], [62], [63], [64], [65], [66], [67], [68], [69], [70], [71], [72], [73], [74], [75], [76], [77], [78], [79], [80], [81], [82].

*S. pseudintermedius* exhibits variable clinical manifestations from superficial infection to invasive infections (Table 4 (Tab. 4)). In dogs *S. pseudintermedius* is mostly associated with skin and soft tissue infection, but in humans it has been reported from various sites, such as the endocardium (endocarditis), ear (otitis externa) and prosthetic joints (infections) [5], [6], [7], [19], [24], [83], [84], [85], [86].

### Drug resistance

In the last decade, phenotypic and automated methods have not been able to differentiate between *S. intermedius* and *S. pseudintermedius*, and the drug resistance pattern is not well studied. Thus, definitive statements cannot be made yet. However, drug resistance in *S. pseudintermedius* has been reported by some authors in veterinary isolates [87]. The highest resistance rates of *Staphylococcus* species are against beta-lactam antibiotics, with almost 95% of the clinical isolates being resistant to penicillin [88], [89], [90]. The resistance mechanism of *S. pseudintermedius* is the same as in* S. aureus*. Drug resistance to beta lactams are mediated by Staphylococcal Chromosomal Cassette (SCCmec). The *mecA* gene is transmitted by plasmids between different *Staphylococcus* species. Methicillin-resistant *Staphylococcus pseudintermedius* (MRSP) in animals has been reported to comprise 67% of total *S. pseudintermedius* infections and in humans it constitutes a prominent risk of drug resistant zoonotic infection transmission. MRSP infections or carriage can occur due to hospitalization, frequent visits to veterinary practices, and use of antimicrobial agents. MRSP can contaminate, colonize or infect animals. Reviews have showed increased resistance in MRSP isolates [17], [91]. Resistance to other classes of antimicrobials are not unusual for *Staphylococcus* genera, and the same is reported for *S. pseudintermedius*. Fluoroquinolones, chloramphenicol, and aminoglycoside (Amikacin) are among the classes which have been reported to be ineffective against MDR isolates. 37% of MRSP clinical isolates in dogs were reported to be resistant to amikacin in the USA [92]. However, screening of drug resistance in *S. pseudintermedius* is an ongoing topic of research, as the isolates are not well-studied in humans. In a case series reported by Somayaji [7], 22.2% of *S. pseudintermedius* human-origin isolates were resistant to methicillin and other classes of antibiotics. The minimum inhibitory concentration (MIC) of almost of all antibiotic classes was much higher in the case of *S. pseudintermedius* biofilm as well as in static conditions, with the exception of Rifampicin, which cannot be always the choice of drug in treatment. Drug resistance in biofilm towards “last-resort” antibiotics such as Vancomycin, Linezolid, Tigecyclin is significant, comparable to biofilm produced by other species in the *Staphylococcus* genus. In hospital settings clinicians might not have any other range of possibilities to treat with antibiotics if this pathogen exhibits higher MIC values to Vancomycin, Linezolid, Tigecyclin than recommended doses as these are the last resort of antibiotics available [59].

### Methicillin-resistant Staphylococcus pseudintermedius (MRSP) screening

Disk-diffusion and broth microdilution tests are the most commonly used phenotypic method for antimicrobial susceptibility testing. For methicillin-resistance screening in *Staphylococcus* species, oxacillin or cefoxitin are used as surrogate markers, because they are sensitive and more stable. In 2018, the Clinical and Laboratory Standards Institute (CLSI document M100-28), with interpretive criteria for the determination of *in vitro* antimicrobial susceptibility of MRSP for isolates from humans, neither the cefoxitin minimum inhibitory concentration (MIC) nor cefoxitin disk tests are reliable for detecting *mecA*-mediated resistance in *S. pseudintermedius*, as they may produce an unacceptably high percentage of false-negative results. This guideline advises that screening for methicillin resistance should be performed by using oxacillin 1 μg disk diffusion or the MIC breakpoints as neither cefoxitin MIC, nor cefoxitin disk tests are reliable for detecting mecA mediated resistance for *S. pseudintermedius* (i.e., resistance in the case of ≥0.5 μg/mL of oxacillin for agar and broth dilution and ≤17 mm for disk diffusion) [93]. PCR targeting the *mecA* gene is the most reliable test for detecting methicillin resistance, but the equipment needed is available in only a few laboratories. The PBP2a latex agglutination test is not reliable and not recommended, as it can result in false-positive results.

### Treatment

The treatment of MRSP is difficult, as there are no pre-existing guidelines or data available on the drug resistance pattern. Animal isolates have shown high prevalence of drug resistance to almost all classes of antibiotics. In European studies [94], [95], animal isolates were screened for genes responsible for drug resistance among different classes of antibiotics; these isolates were found to be positive for all genes similar to those found in *S. aureus*. The high level of drug resistance in *S. pseudintermedius* limits treatment options. Three of 24 strains were diagnosed as MRSP in a study by Somayaji et al [7]. There was no specific treatment for these patients, who were managed as outpatients. The pattern showed resistance against antimicrobial classes such as macrolides, sulfonamides, and fluoroquinolones [7]. Decolonization in animals may be achieved by with products containing chlorhexidine. Since the MRSP infections exhibit a wide spectrum of clinical symptoms and manifestations, a structured, tailored treatment plan is required, taking into account severity of disease, comorbid conditions and hospitalization.

The resistance to antibiotics is directly proportional to antibiotic use. This is the point where clinicians have more control. To prevent further antibiotic resistance, the European Wound Management Association released position document which emphasize providing an optimal environment to promote rapid healing, restricting antibiotic use to situations where they are specifically indicated, and appropriate use to reduce antibiotic resistance[96]. Certain critical antibiotics for the treatment of MRSA in humans, such as mupirocin, are legally restricted to animals in some European countries [97]. 90% of dogs who underwent treatment for MRSP, also showed resistant to antibiotic classes approved for use in humans (ciprofloxacin, clindamycin, erythromycin, kanamycin, streptomycin, and trimethoprim). Rifampicin resistance was observed in 9 out of 10 *S. pseudintermedius* isolates [97], [92]. Frequent use of these drugs can increase the risk of developing antibiotic resistance, thus minimizing treatment options even for human cases.

## Conclusions

Although *S. pseudintermedius* is a known colonizer in dogs, its sudden emergence in humans is cause for concern [5]. Since the literature suggests invasive infections occurred in humans, *S. pseudintermedius* certainly has the potential to be virulent in human hosts [5], [7], [24], [84], [88]. It is associated with implant, skin and wound infections. Correct identification with use of rapid, easy-to-use tools is required to produce a large database for future studies and establish management guidelines for infections caused by *S. pseudintermedius*. As data is still lacking, conclusion about its pathogenesis are not yet possible, so that all potentially pathogenic factors should be monitored before starting treatment. Both *in vitro* and *in vivo* studies are needed to establish the connection between virulence factors. From a microbiological perspective, all possible methods should be used to differentiate *S. pseudintermedius* from SIG. and drug resistance patterns need to be documented for future studies. More research is required to identify and establish the link between pathogenesis and clinical disease caused by *S. pseudintermedius* correlation in case of human. Strict indications should apply when using antibiotics to treat animals. Treatment of *S. pseudintermedius* in animals is very critical, as there are no guidelines yet on using human antibiotics for treatment, which might increase the drug resistance level in these strains. So far its colonization has not been reported from humans, but it is advisable to screen patients for this pathogen when coagulase-positive species of *Staphylococcus* are isolated, especially from wound or skin infections. There is urgent need to establish guidelines to treat animals so the emergence of drug resistance can be stopped.

## Notes

### Competing interests

The authors declare that they have no competing interests.

## Figures and Tables

**Table 1:**
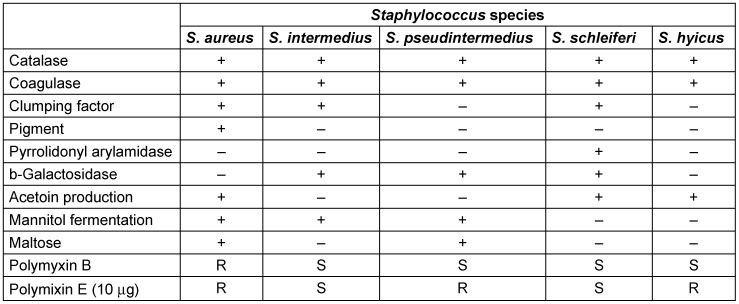
Phenotypic tests for differentiation of coagulase-positive *Staphylococcus* species

**Table 2:**
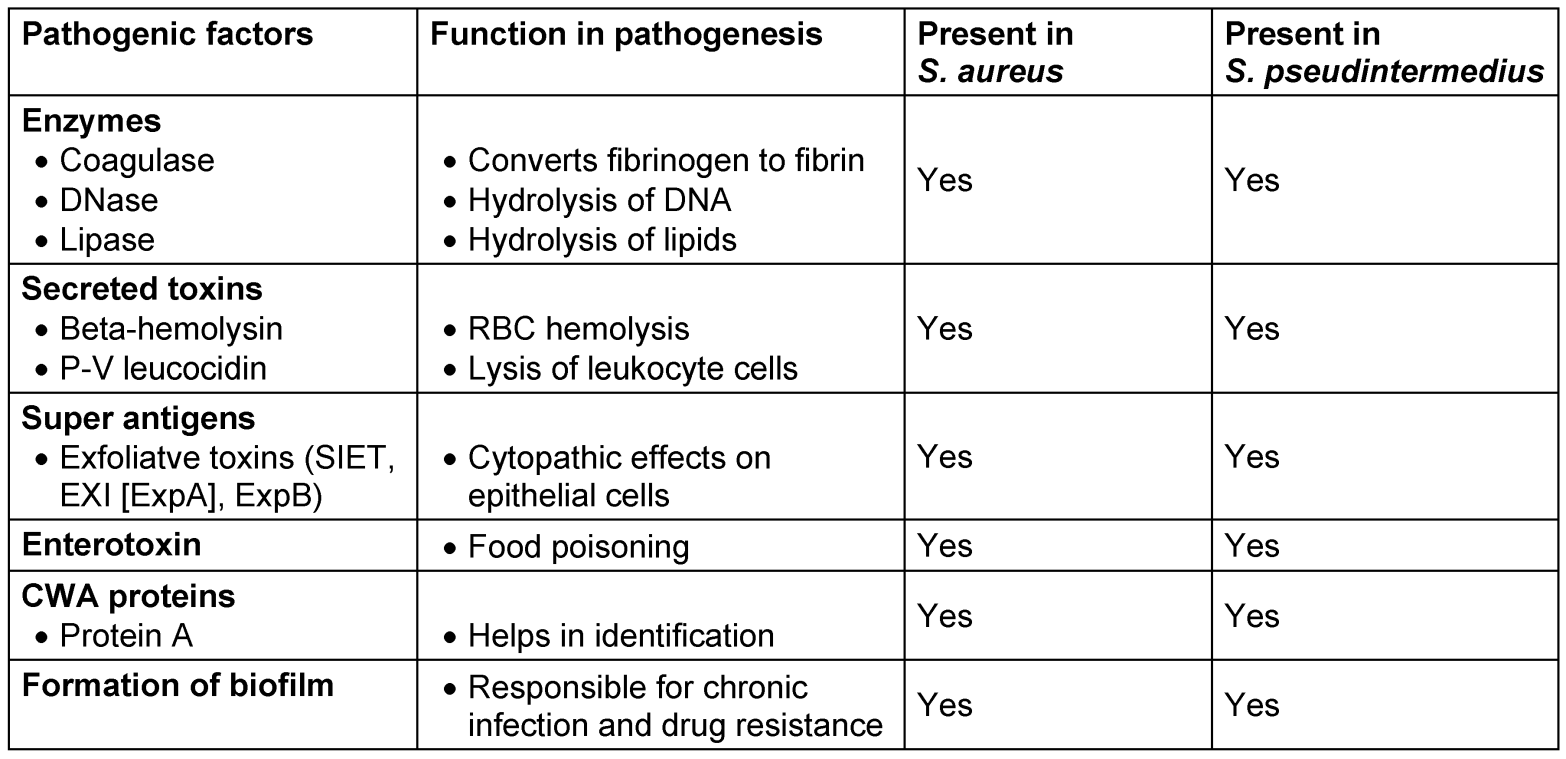
Resemblance of pathogenic factors in *S. aureus* and *S. pseudintermedius*

**Table 3:**
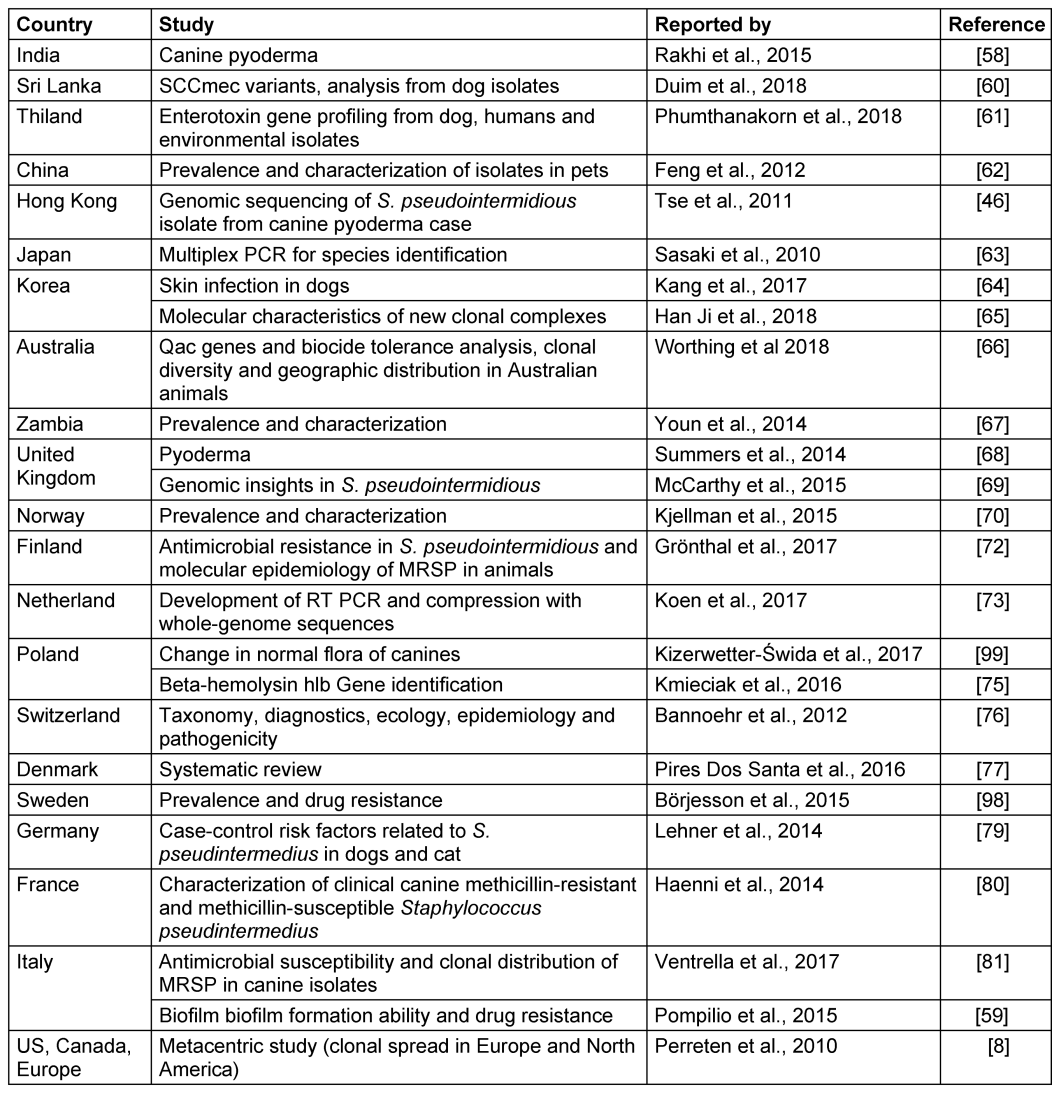
Type of study and country reported

**Table 4:**
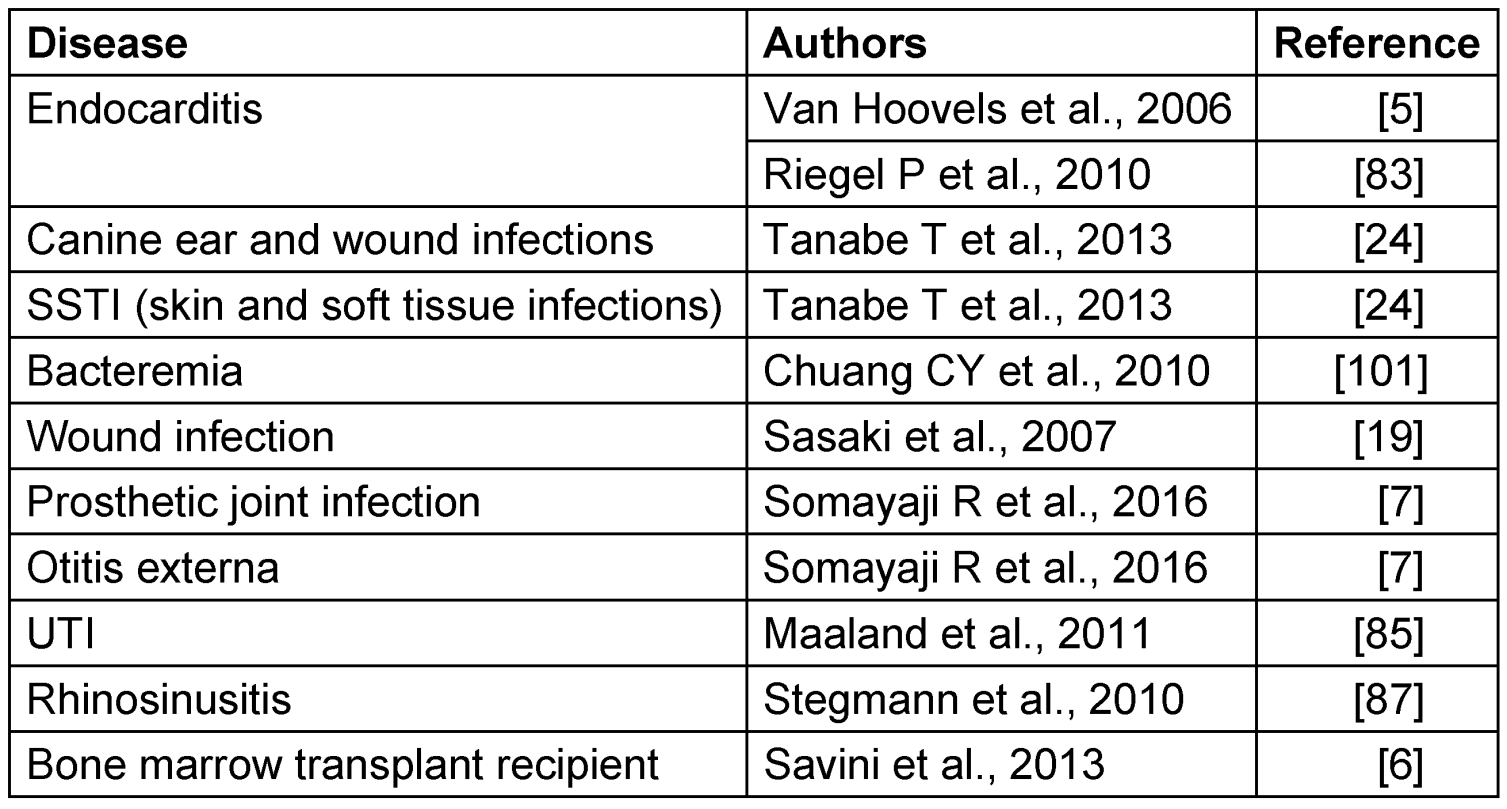
Clinical diseases caused in humans and dogs
